# Interlayer-Tailored Alkyl-MXenes for Selective Electrochemical Lithium-Ion Extraction

**DOI:** 10.1021/acsenergylett.5c03009

**Published:** 2025-12-12

**Authors:** Cansu Kök, Karamullah Eisawi, Jean G. A. Ruthes, Burcu Tan, Antje Quade, Michael Naguib, Volker Presser

**Affiliations:** † INM - Leibniz Institute for New Materials, D2 2, 66123 Saarbrücken, Germany; ‡ Department of Materials Science & Engineering, Saarland University, Campus D2 2, 66123 Saarbrücken, Germany; § Department of Physics and Engineering Physics, Tulane University, New Orleans, Louisiana 70118, United States of America; ∥ 28372 Leibniz Institute for Plasma Science and Technology (INP), Felix-Hausdorff-Straße 2, 17489 Greifswald, Germany; ⊥ saarene, Saarland Center for Energy Materials and Sustainability, Campus C4 2, 66123 Saarbrücken, Germany

## Abstract

The efficient and selective extraction of lithium ions from aqueous media is crucial for resource recovery, yet remains challenging due to the chemical similarity of coexisting alkali ions, such as sodium. In this study, we report a two-step electrochemical strategy that utilizes tailored MXene electrodes for lithium ion extraction with enhanced selectivity and extraction rates. By preintercalating hexadecylamine (HDA) and decyltrimethylammonium (C10), which are long-chain organic molecules, into the Ti_3_C_2_T_*x*_ MXene structure, we tailored the interlayer environment to favor lithium ions over sodium ions. The HDA-intercalated MXene demonstrated high Li^+^/Na^+^ selectivity with a lithium ion uptake of 2.2 mmol/L and a suppressed sodium ion uptake (<0.2 mmol/L). Extended cycling revealed that molecular preintercalation modulates ion transport pathways and influences structural and electrochemical stability. Both HDA-Ti_3_C_2_T_*x*_ and C10-Ti_3_C_2_T_*x*_ maintained a lithium ion purity of nearly 100% over 50 cycles.

## Graphical abstract

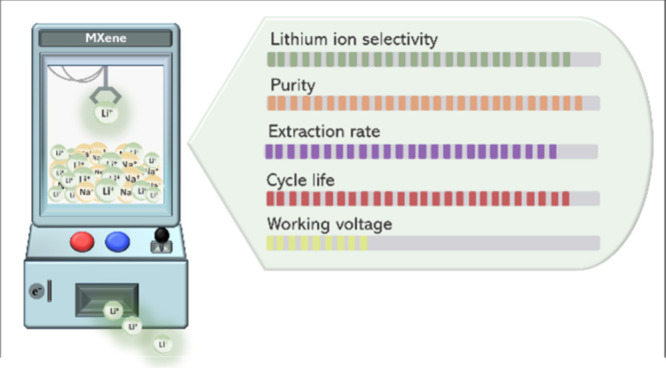

The increasing demand for lithium requires efficient and sustainable extraction technologies.^1^ Conventional approaches, such as evaporation ponds and chemical precipitation, are limited by their inefficiency, high energy consumption, and environmental concerns.^2^ As a promising alternative, electrochemical lithium ion separation offers high selectivity, low environmental impact, and scalability.^3–5^ MXenes, which are two-dimensional transition metal carbides and nitrides, have attracted attention owing to their high electrical conductivity, surface chemistry tunability, and high affinity for cations.^6,7^ Their layered structure with surface functional groups enables the precise tuning of their electrochemical properties for lithium-selective separation.^8,9^ Furthermore, their high electrical conductivity ensures high charge/discharge rates with mechanical and chemical stability for prolonged electrochemical operations.^10^ Recent studies have demonstrated the potential of MXenes in Li-selective electrosorption, revealing high capacity, fast kinetics, and high lithium ion diffusion mobility compared to traditional electrode materials.^11^ Strategies such as doping, defect engineering, adjustable interlayer spacing, and composite formation with other nanomaterials can further enhance their lithium selectivity and durability, rendering MXenes a promising platform for sustainable extraction technologies.^12,13^

By modulating surface terminations, the interlayer spacing for ion sieving, and electronic characteristics, MXene-based electrodes can achieve enhanced lithium ion adsorption and desorption, thereby facilitating the selective separation of lithium ions from complex brine solutions.^14–17^ For example, Chen et al.^8^ developed a hybrid membrane by immobilizing a titanium-based lithium ion sieve (HTO) on a polyvinyl chloride (PVC) film and used MXene to enhance its adsorption sites. The resulting HTO/MXene@PVC membrane demonstrated good lithium ion adsorption with a maximum capacity of 25.4 mg/g. Ge et al. proposed a MXene-based composite membrane by incorporating poly­(sodium 4-styrenesulfonate) into MXene for Li^+^/Mg^2+^ separation.^15^ Abdelrahman et al. reported on hydrogel nanocomposite sorbents containing sulfonated graphene oxide (SGO), MXene, and alginate.^16^ The sulfonic acid groups on SGO enable selective lithium ion binding by facilitating Li^+^/H^+^ exchange while minimizing interference from other ions. Wang et al. fabricated a flexible hybrid film by embedding MnO_2_·0.5H_2_O nanoparticles into a 2D MXene/1D carbon nanofiber structure. The material facilitated rapid lithium ion transport and enhanced lithium ion selectivity by blocking interfering ions through steric hindrance.^17^ In particular, it achieved a sorption capacity of 21.4 mg/g, and lithium ion selectivity (e.g., Li/Na = 29,232 and Li/Mg = 3,606).^17^

Ren et al.^18^ employed a hydrogen bond interaction strategy to integrate 2D MXenes as interfacial agents between Li_1.33_Mn_1.67_O_4_ and polysulfone, forming a composite membrane. Their design achieved a high lithium sorption capacity of 21.1 mg/g and stable cycling performance. In 2024, Fahem et al.^13^ recovered lithium ions through electrosorption using pseudocapacitive electrodes. Composite materials of polystyrenesulfonated MXene (PM) and a sodium titanate/graphene oxide (NG) were synthesized and applied in a single-cell capacitive deionization system.^13^ PM and NG electrodes tested in binary, ternary, and quaternary ionic solutions achieved a lithium ion recovery purity ranging from 59% to 96% and 60% to 77%, respectively.^13^ The intercalation of alkylammonium ions into the MXene layers effectively modulates their interlayer spacing and surface chemistry, thereby unlocking new application opportunities for MXenes.^19^ Particularly, alkylammonium ion preintercalation leads to the formation of pillars that expand the interlayer spacing, enabling the intercalation of large room-temperature ionic liquid cations and resulting in high energy and power densities in MXene-based supercapacitors.^20^

Although MXene and MXene-based materials exhibit significant potential for lithium ion recovery, research in this area remains limited. To further advance the understanding and efficiency of selective electrochemical lithium ion recovery, our study explored a range of MXene-based materials, with a focus on tunable interlayer spacing. By controlling the interlayer structure, the selective uptake of lithium ions was optimized to enhance the selectivity and overall extraction performance for lithium ion recovery.

Our work highlights the advantages of modifying MXenes with organic molecules for lithium ion extraction. Unlike electrode-level strategies that primarily enhance ion pathways or electrode densities, organic intercalation enables the direct, molecular-level control of interlayer spacing, surface chemistry, and electrostatic environments.^21^ This creates selective channels that facilitate lithium ion transport, linking fundamental insights into ion sieving with practical performance and positioning organic-modified MXenes as a highly promising ion-selective material. As illustrated in Figure 1A, a mixture of HF and LiCl was used during the etching process to simultaneously remove Al and intercalate lithium ions between the Ti_3_C_2_T_*x*_ layers. Following etching, cation exchange was achieved by replacing lithium ions with ammonium salts or alkylamines, resulting in an increased interlayer spacing due to the incorporation of long alkyl chains. Figures 1B–J show the morphologies of the multilayered structure of pristine Ti_3_C_2_T_*x*_, C10-Ti_3_C_2_T_*x*_, and HDA-Ti_3_C_2_T_*x*_. X-ray diffractograms of the pristine Ti_3_C_2_T_*x*_, C10-Ti_3_C_2_T_*x*_, and HDA-Ti_3_C_2_T_*x*_ are presented in Figure 1D, where a progressive shift of the (002) reflection to lower angles was observed with increasing intercalant chain length, confirming the expansion of the interlayer spacing (Figure 1E).

**Figure 1:**
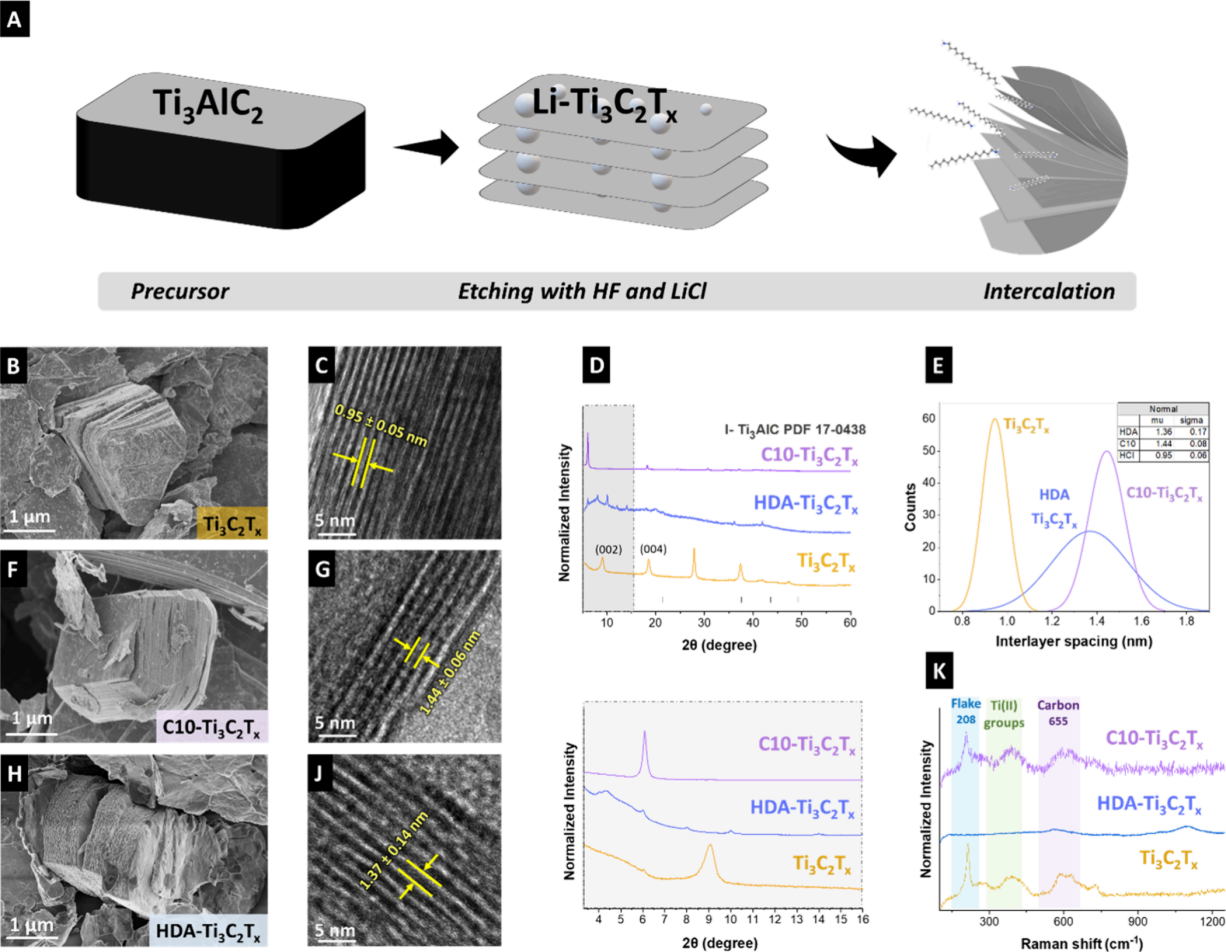
(A) Schematic representation of the synthesis of the MXene. (B–K) Material characterization of Ti_3_C_2_T_*x*_, C10-Ti_3_C_2_T_*x*_, and HDA-Ti_3_C_2_T_*x*_. (B,F,H) Scanning electron micrographs of the materials. (C,G,J) Transmission electron micrographs, (D) X-ray diffractograms. (E) Interlayer spacing values of the materials measured via transmission electron microscopy. ( K) Raman spectra.

Raman spectroscopy showed the presence of the D-band and G-band of incompletely graphitic carbon (Figure 1K).^22^ The characteristic signals at 215 cm^–1^, 394 cm^–1^, and 605 cm^–1^ correspond to out-of-plane (A_1g_) and in-plane (E_g_) shifts, characteristics of Ti_3_C_2_T_*x*_ MXene flakes, documenting Al removal from the initial MAX phase and the successful synthesis of MXene.^23^ The shifts at 394 cm^–1^ were attributed to the E_g_ of Ti atoms, and the bands at 605 cm^–1^ were in the range of carbon vibrations (A_1g_-E_g_).^24^

X-ray photoelectron survey spectra showed the presence of O, F, Ti, and C at the surface of the samples (Supporting Information, Figure S1). The C10-Ti_3_C_2_T_*x*_ and HDA-Ti_3_C_2_T_*x*_ samples contained additional N and only traces of Cl (Figure 2F). In addition to aliphatically and hydroxidically bound carbon, all C 1s signals showed a distinct peak at a lower binding energy of 281.8 eV, which could be assigned to the carbidic bond (Figure 2A). The highly resolved Ti 2p spectra were fitted with four doublets corresponding to the Ti 2p_3/2_ and Ti 2p_1/2_ states in Ti-C with different surface terminations (C-Ti-T_O_, C-Ti-T_F,O_, C-Ti-T_F_), and TiO_2_ surroundings, respectively.^25–27^ A Shirley background was selected for all peaks. Owing to their conductivity, asymmetric line shapes were employed for the MXene components, whereas a symmetric line shape was used for the oxide.^25^ The energetic difference ΔTi 2p, which was due to spin–orbital splitting, was set to 6.1 eV for the MXene components. For the oxide component, ΔTi 2p was set to 5.6 eV.^25,27^

**Figure 2:**
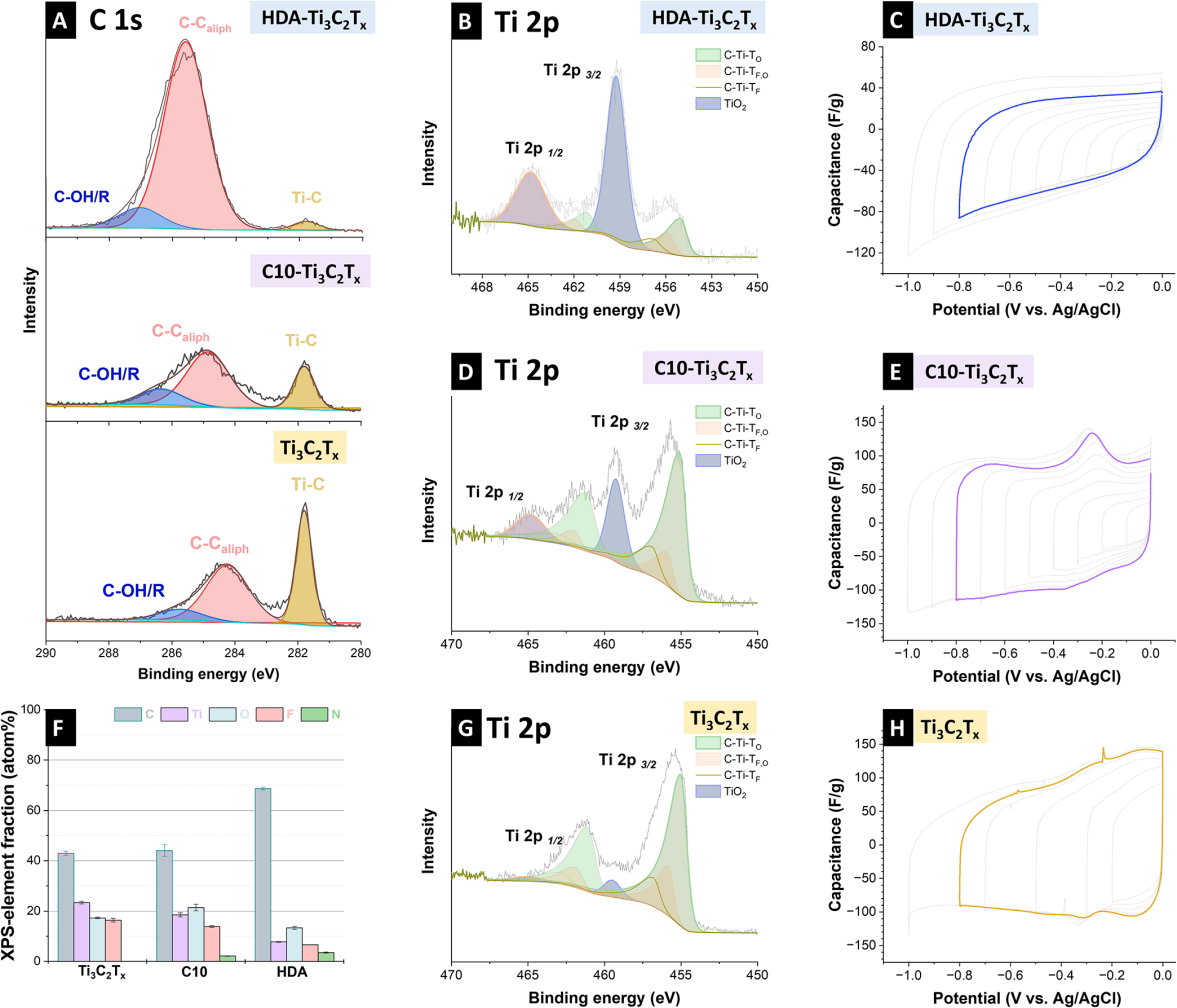
(A-H) Chemical composition and cyclic voltammograms of HDA-Ti_3_C_2_T_*x*_, C10-Ti_3_C_2_T_*x*_, Ti_3_C_2_T_*x*_. (A) Comparison of X-ray photoelectron C 1s spectra. (B,D,G) Highly resolved measured Ti 2p peaks. (F) XPS data-derived elemental composition. (C,E,H) Electrochemical window opening via cyclic voltammetry.

The Ti 2p_3/2_ peak at 459.2 eV was attributed to TiO_2_ species. The peaks located at 455.1, 455.9, and 456.9 eV were assigned to C-Ti bonded to O (C-Ti-T_O_), C-Ti bonded to both F and O (C-Ti-T_F,O_), and C-Ti bonded only to F (C-Ti-T_F_) (Figure 2B-D,G).^28^ The Ti 2p spectrum of the pristine sample exhibited the highest carbide content among the analyzed samples (Figure 2G). In contrast, HDA-Ti_3_C_2_T_*x*_ displayed the lowest carbide contribution and the most pronounced presence of TiO_2_ (Figure 2B), suggesting that partial surface oxidation occurred during synthesis (Supporting Information, Figure S2). This oxidation was likely facilitated by the relatively elevated synthesis temperature (75 °C) employed in the preparation process of HDA-Ti_3_C_2_T_*x*_.

The N 1s spectra (Supporting Information, Figure S3) provided direct evidence of N-containing species in the C10-Ti_3_C_2_T_*x*_ and HDA-Ti_3_C_2_T_*x*_ samples. The absence of any peaks at 396.5–396.8 eV corresponding to Ti–N confirmed that these N-species were intercalated rather than covalently bonded terminations of the MXene.^29^ For C10-Ti_3_C_2_T_*x*_, the N 1s at 402.1 eV corresponded to trimethylammonium group −N^+^(CH_3_)_3_. For HDA-Ti_3_C_2_T_*x*_, the additional peak at 401 eV could be attributed to protonated amine species R-NH_3_^+^.^30^ Therefore, both C10 and HDA intercalated as positively charged organic ions, electrostatically interacting with the negatively charged surface of the MXenes while displacing the lithium ions originally present between the MXene layers.^19,31^ Evidently, the long alkyl chains stabilized the expanded interlayer spacing, which was consistent with the structural changes observed in the X-ray diffractograms.

Comparative electrochemical data demonstrated that the surface modifications of Ti_3_C_2_T_*x*_ MXene materials influenced their ion selectivity (Figure 2C,E,H). In 1 M NaCl, HDA-Ti_3_C_2_T_*x*_ and C10-Ti_3_C_2_T_*x*_ exhibited higher current responses across various scan rates, indicating faster kinetics than that of pristine Ti_3_C_2_T_*x*_, which showed lower and less symmetric cyclic voltammograms (Supporting Information, Figure S4). In 1 M LiCl, all the samples display increased current densities relative to NaCl, with C10-Ti_3_C_2_T_*x*_ showing prominent redox features, indicating favorable lithium ion intercalation.

The cyclic voltammograms revealed that HDA-Ti_3_C_2_T_*x*_ and C10-Ti_3_C_2_T_*x*_ enhanced lithium ion intercalation over sodium ions in different electrolytes at 1 mV/s, while pristine Ti_3_C_2_T_*x*_ demonstrated a stronger sodium ion response (Supporting Information, Figure S4G-J). This selectivity was attributed to the electrostatic and coordination interactions between Li ions and the functional groups of the alkyl chains. Owing to their higher charge density and stronger Lewis acidity compared to sodium ions, lithium ions can establish more stable ion-dipole interactions with electron-rich sites along the alkyl chains. In contrast, such interactions are energetically less favorable for sodium ions.^32^ The specific capacitance of the three MXene electrodes was evaluated at a scan rate of 1 mV/s over a 0–0.8 V window in 1 M LiCl (Supporting Information, Table S1). HDA-Ti_3_C_2_T_*x*_ and C10-Ti_3_C_2_T_*x*_ yielded capacitances of 37 and 54 F/g, respectively, while the Ti_3_C_2_T_*x*_ sample showed an intermediate capacitance of 23 F/g. Thus, the surface modification and synthesis approach significantly influenced the electrochemical performance.

The two-step lithium ion extraction process is illustrated in Figure 3A. In the first step, the application of an external potential drove lithium ion intercalation while rejecting sodium ions and reversing the potential released lithium ions back into the solution. Figure 3B-D presents the variations in the lithium ion and sodium ion concentrations observed for the different MXene-based electrodes over 50 cycles. HDA-Ti_3_C_2_T_*x*_ stably extracted lithium ions with minimal sodium ion uptake (2.2 mmol/L and 0.18 mmol/L, respectively) (Figure 3B-E). A comparable trend was observed for C10-Ti_3_C_2_T_*x*_; however, a small degree of sodium ion uptake was detected (2.7 mmol/L and 0.4 mmol/L, respectively) (Figure 3C-F). In contrast, the pristine electrode exhibited unstable cycling and the highest lithium ion uptake (3.3 mmol/L) (Figure 3D-G) but with significant sodium ion uptake (1.1 mmol/L), likely due to insufficient control over the interlayer spacing.

**Figure 3:**
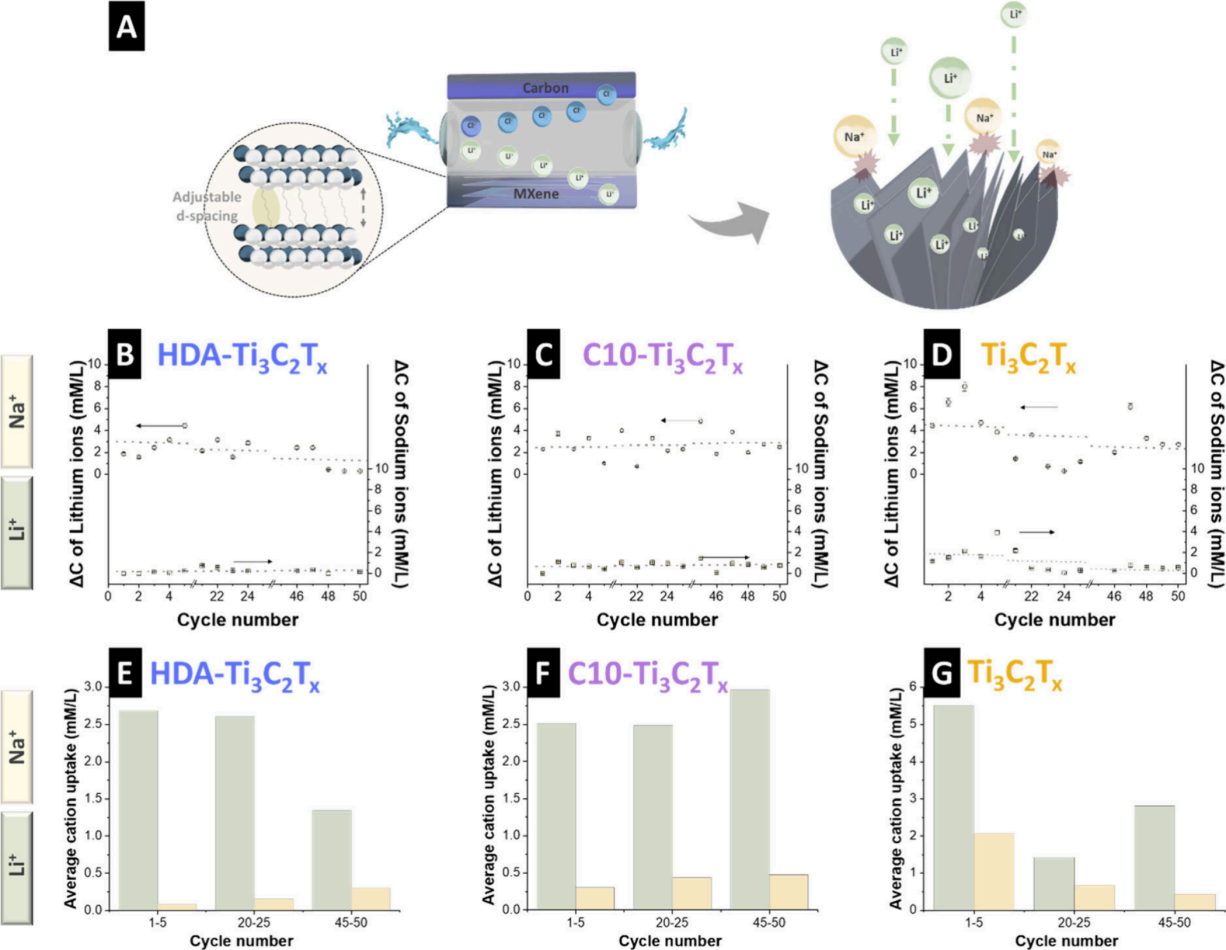
(A) Schematic representation of the electrochemical lithium ion extraction system. Cation uptake of the electrodes for (B) HDA-Ti_3_C_2_T_*x*_, (C) C10-Ti_3_C_2_T_*x*_, and (D) Ti_3_C_2_T_*x*_. Average cation uptake of the electrodes for (E) HDA-Ti_3_C_2_T_*x*_, (F) C10-Ti_3_C_2_T_*x*_, and (G) pristine Ti_3_C_2_T_*x*_.

Although pristine Ti_3_C_2_T_*x*_ exhibited the highest lithium ion intercalation capacity, it also demonstrated lower selectivity, as evidenced by its substantial sodium ion uptake. Thus, expanding the interlayer spacing through molecular intercalation significantly enhanced the lithium ion selectivity. Among the tested electrodes, HDA-Ti_3_C_2_T_*x*_ and C10-Ti_3_C_2_T_*x*_ exhibited the best performance, combining a high Li-ion uptake with pronounced Na-ion exclusion. These results indicate a compatibility threshold between the solvated ionic diameter and interlayer spacing of the layered materials.^32^ Pristine Ti_3_C_2_T_*x*_, with a relatively narrow interlayer spacing of 0.95 nm (9.5 Å), likely restricted the intercalation of solvated ions, thereby limiting the uptake and desorption performance. In contrast, HDA-Ti_3_C_2_T_*x*_ and C10-Ti_3_C_2_T_*x*_, with larger interlayer spacings (1.37 and 1.44 nm, respectively), were more accommodating to solvated ions, potentially enabling more efficient ion diffusion and transport (Figure 1E).

The HDA-Ti_3_C_2_T_*x*_, C10-Ti_3_C_2_T_*x*_, and pristine Ti_3_C_2_T_*x*_ materials showed high Li^+^-extraction rates of 207 μm/cm^2^/h, 235 μm/cm^2^/h, and 310 μm/cm^2^/h, respectively (Figure 4A). Although Ti_3_C_2_T_*x*_ had a high initial extraction rate during the first five cycles, its performance declined between cycles 20 and 25, likely because of its unmodified nature. In contrast, the extraction rates of HDA-Ti_3_C_2_T_*x*_ and C10-Ti_3_C_2_T_*x*_ remained relatively stable.

**Figure 4:**
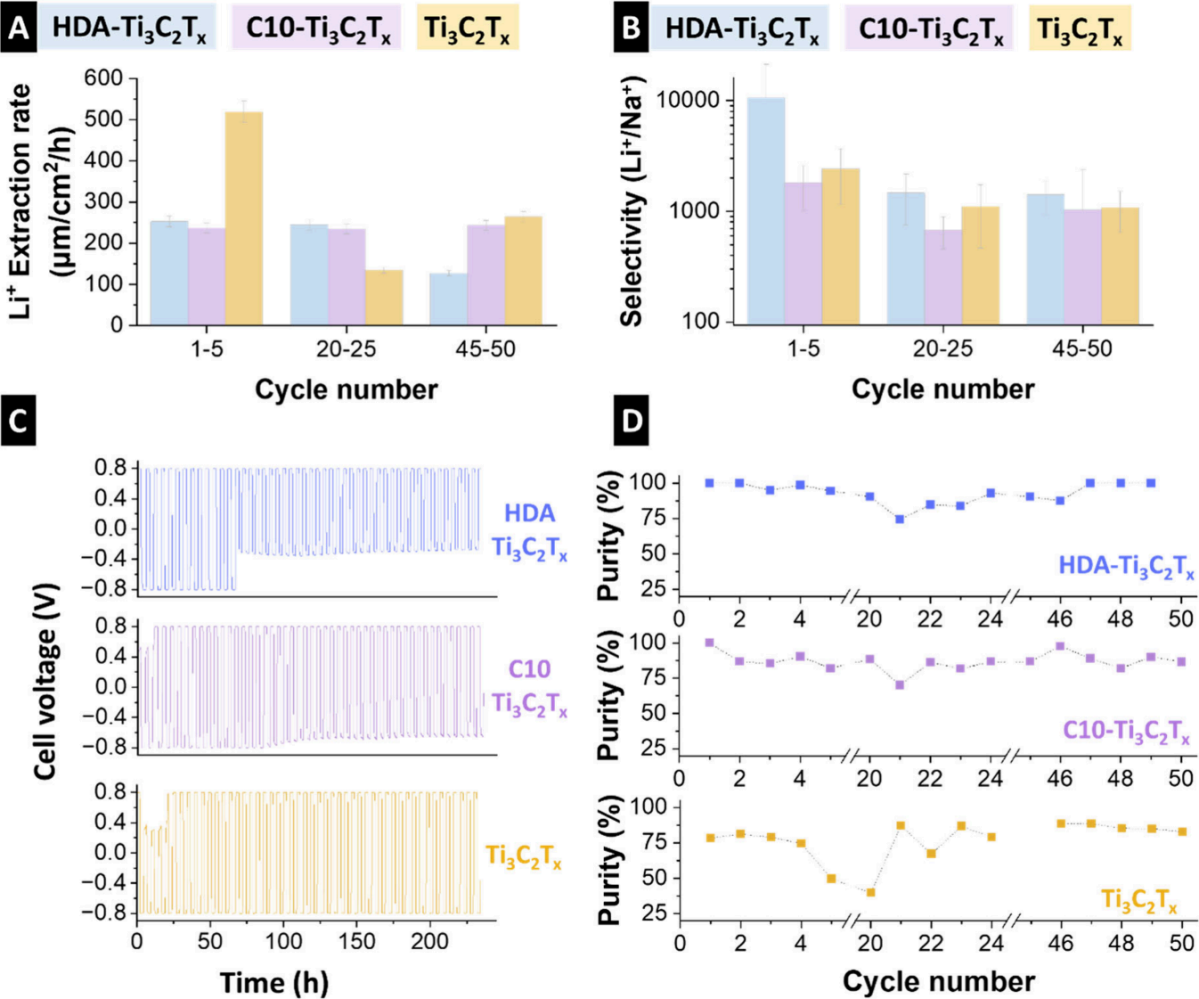
(A) Lithium ion extraction rate of the electrodes. (B) Lithium ion vs sodium ion selectivity of the electrodes. (C) Stability test of the electrodes for operation within −0.8 V to +0.8 V. (D) Purity of the recovery solution.

All electrodes exhibited a higher initial Li^+^/Na^+^ selectivity (Figure 4B) that decreased over time. HDA-Ti_3_C_2_T_*x*_ exhibited the highest early cycle (∼10,600) and late-cycle (∼1,400) selectivities, confirming the effectiveness of HDA intercalation in favoring Li ions. For comparison, the late-cycle (i.e., cycles 45**–**50) Li^+^/Na^+^ selectivities of C10-Ti_3_C_2_T_*x*_ and pristine Ti_3_C_2_T_*x*_ were lower (∼1,000). The relatively high selectivity of pristine Ti_3_C_2_T_*x*_ is attributed to the LiCl treatment during synthesis, which preloads lithium ions into the interlayer galleries, effectively templating the structure and lowering the energy barrier for lithium ion insertion or exchange compared to other ions.^33–36^ Post-mortem SEM confirmed structural degradation after 50 cycles, particularly for pristine Ti_3_C_2_T_*x*_, where the intercalated structure was no longer visible (Supporting Information, Figure S5). The absence of distinct X-ray reflections may indicate a significant expansion in the interlayer spacing beyond the detection limit of XRD used in this study. Alternatively, this could reflect the structural degradation and amorphization of MXene (Supporting Information, Figure S5).^37^ The measured increase in the lithium ion extraction rate of pristine Ti_3_C_2_T_*x*_ during cycles 45–50 may be attributed to electrochemically induced interlayer expansion over cycling. Unlike preintercalated materials, which initially benefit from expanded spacing, pristine Ti_3_C_2_T_*x*_ appeared to undergo progressive structural activation, likely owing to gradual Li^+^/solvent intercalation. This late-stage expansion enhanced the lithium ion mobility and temporarily improved the purity and extraction rates.

To investigate this phenomenon, we evaluated the electrode stability through extended charge–discharge cycling with the aim of elucidating the correlation between structural degradation and selectivity loss over time (Figure 4C). HDA-Ti_3_C_2_T_*x*_ exhibited a higher degradation rate than the other electrodes. Despite the tailoring effect of long-chain alkylamines, such as HDA, on the MXene structure, their incorporation may introduce trade-offs, including a reduced electrical conductivity, hindered ion transport, and limited electrochemical accessibility owing to the insulating and sterically bulky nature of the organic chains.^7,38^ Degradation may also occur by the action of bulk aqueous electrolytes on the interlayer spacing, not just ions. Such a continuous exposure of the interlayer spacing to the aqueous electrolyte may accelerate MXene degradation.

A key challenge in Li extraction is maximizing both the rate and purity, because faster methods often reduce selectivity. The MXene electrodes exhibited both high purity and promising Li-ion extraction capacity (Figure 4D). HDA-Ti_3_C_2_T_*x*_ and C10-Ti_3_C_2_T_*x*_ maintained nearly 100% purity after 50 cycles, whereas the purity of pristine Ti_3_C_2_T_*x*_ significantly decreased to 30% with an unstable performance.

HDA-Ti_3_C_2_T_*x*_ and C10-Ti_3_C_2_T_*x*_ exhibited an average Li^+^/Na^+^ selectivity of approximately 1500, which is competitive with that of advanced lithium ion selective materials (Table 1). Lithium superionic conductors achieve a higher selectivity (10^4^–10^6^) but face flux and scalability challenges.^39–41^ Diffusion-based methods and LFP electrodes report similar selectivity (900–1000), but with limited recovery or cycling stability.^5^ LiMn_2_O_4_ exhibits high diffusion coefficients (10^–9^–10^–11^ cm^2^·s^–1^), structural stability, and low cost, with optimized electrodes reaching selectivity near 1000.^42^ Manganese-based adsorbents also show substantial selectivity (Li^+^/Na^+^ = 371) in sodium-rich wastewater.^43^ Although the HDA-Ti_3_C_2_T_*x*_ selectivity decreased with time, its initial performance rivals or exceeds that of leading membranes and adsorbents, underscoring its potential for practical lithium extraction where both selectivity and durability are important.

**Table 1:** Lithium-Ion Selectivity Values in This Study Compared to Existing Literature on Advanced Materials

Material	Lithium ion selectivity component	Lithium ion selectivity	Reference
M-T-LIS	adsorbent	674	^45^
λ-MnO_2_	adsorbent	3.4 × 10^4^	^46^
12-Crown-4 ether	membrane	(5–1.0) × 10^2^	^47^
Spiropyran crown ether	membrane	1.0 × 10^3^	^48^
Polyamide (PA)	membrane	4.0 × 10^3^	^49^
LiTi_2_(PO_4_)_3_	membrane	ca. 2.6 × 10^4^	^39^
Li_1+*x*_Al_*x*_Ge_2–*x*_(PO_4_)_3_	membrane	4.1 × 10^5^	^5^
Li_0.33_La_0.56_TiO_3_ (LLTO)	membrane	4.5 × 10^7^	^50^
LiMn_2_O_4_/Li_1*–x*_Mn_2_O_4_	electrode	1.4 × 10^3^	^42^
TiO_2_-coated FePO_4_	electrode	1.8 × 10^4^	^40^
MXene (HDA-Ti_3_C_2_T_*x*_)	electrode	av. 4.5 × 10^3^	This work

This study elucidates the efficacy of interlayer engineering in MXene-based electrodes for the selective extraction of lithium ions using an electrochemical cycling process. The tailored MXenes, particularly those preintercalated with HDA, exhibited an excellent combination of high lithium ion uptake and high sodium ion rejection, outperforming their C10-Ti_3_C_2_T_*x*_ and pristine Ti_3_C_2_T_*x*_ counterparts. The Li^+^/Na^+^ selectivity observed for HDA-Ti_3_C_2_T_*x*_ highlights the role of alkyl chains in modulating the interlayer chemistry and enhancing ion sieving. Pristine Ti_3_C_2_T_*x*_ exhibited the highest lithium ion uptake but lower selectivity and stability. Long-term cycling revealed a stable performance for C10-Ti_3_C_2_T_*x*_. Conversely, structural degradation was observed for HDA-Ti_3_C_2_T_*x*_, which was attributed to the oxidative instability of bulky organic chains that compromised the electrochemical accessibility and transport kinetics. HDA-Ti_3_C_2_T_*x*_ and C10-Ti_3_C_2_T_*x*_ retained lithium ion purity levels approaching 100% over 50 cycles, in contrast to the 30% decrease observed for pristine MXene. Therefore, interlayer functionalization is an effective strategy for advancing MXene electrodes toward high-rate, high-purity lithium ion extraction, thereby bridging the gap between ion selectivity and operational stability in aqueous electrochemical systems. This way, lithium ion harvesting from natural media^4^ or within the context of lithium-ion battery recycling^44^ may enable circular applications.^39^

## Supplementary Material



## Data Availability

The data can be made available upon request.

## Notes

Experimental description, details on the material synthesis, XPS survey spectra of the electrodes, *post-mortem* SEM of the electrodes, *post-mortem* XRD of the electrodes, data obtained from cyclic voltammetry, and key performance metrics of the electrodes (PDF)
