# Supplementary material for: Interlayer-Tailored Alkyl-MXenes for Selective Electrochemical Lithium-Ion Extraction
Source: ACS Energy Lett. 2025 Dec 12;11(1):508–16. doi: 10.1021/acsenergylett.5c03009 (PMC12797855; doi:10.1021/acsenergylett.5c03009)
Supplement: Supplementary file 1 [file nz5c03009_si_001.pdf]

Supporting Information

Interlayer-tailored alkyl-MXenes for selective electrochemical lithium-ion extraction

Cansu Kök,[1,2] Karamullah Eisawi,[3] Jean G. A. Ruthes,[1,2] Burcu Tan,[1,2] Antje Quade,[4] Michael Naguib,[3] and Volker Presser,[1,2,5,]*

1 *INM - Leibniz Institute for New Materials, D2 2, 66123, Saarbrücken, Germany*

2 *Department of Materials Science & Engineering, Saarland University, Campus D2 2, 66123, Saarbrücken, Germany*

3 *Department of Physics and Engineering Physics, Tulane University, New Orleans, LA 70118, United States of America*

4 *Leibniz Institute for Plasma Science and Technology (INP), Felix-Hausdorff-Straße 2, 17489 Greifswald, Germany*

5 *saarene, Saarland Center for Energy Materials and Sustainability, Campus C4 2, 66123 Saarbrücken, Germany*

* Corresponding author's email: volker.presser@leibniz-inm.de

Experimental description

Synthesis of the Ti_3AlC_2 MAX phase

Ti_3AlC_2 was synthesized using powders of titanium (-325 mesh, 99%, Thermo Scientific), aluminum (-325 mesh, 99.5%, Thermo Scientific), and graphite (7-11 µm, 99%, Alfa Aesar) in a molar ratio of 3.00:1.20:1.88. The powders were loaded into a high-density polyethylene (HDPE) bottle along with ten yttria-stabilized zirconia balls (10 mm diameter), then mixed using a Turbula T2F mixer at 56 rpm for 3 h. The resulting mixture was transferred to an alumina crucible and placed at the center of an alumina tube furnace. The crucible was heated to 1600 °C for 2 h under continuous argon flow (0.2 L/min), at a ramp rate of 5 °C/min. After thermal treatment, the furnace was allowed to cool naturally to room temperature under an argon atmosphere. The synthesized product, a lightly sintered Ti_3AlC_2 brick, was ground and sieved to obtain particles smaller than 45 µm.

Synthesis of different alkylamine-based intercalated $Ti_3C_2T_x$ MXene

$Ti_3C_2T_x$ was synthesized by selective etching of Al from Ti_3AlC_2. Ti_3AlC_2 powder (1 g) was slowly added to a 50 mL solution containing 10 mass% hydrofluoric acid (HF, 48-51%, Acros Organics) and 1 g of lithium chloride (LiCl, 99%, Thermo Scientific) in an HDPE bottle, which was stirred at room temperature for 24 h.(1, 2) The resulting etched powder was then divided into 50 mL centrifuge tubes at a ratio equivalent to 0.5 g of the starting Ti_3AlC_2 per tube. To remove excess lithium ions and facilitate subsequent cation exchange, the powders were washed four times with 6 M hydrochloric acid (HCl, 37%, Fischer chemicals), followed by two washes with deionized water to remove residual acid. The protonated wet sediment was either vacuum-filtered to obtain a pristine $Ti_3C_2T_x$ sample or retained in its wet form for further AA intercalation. Alkylamine-based intercalated $Ti_3C_2T_x$ MXenes were prepared using a wet chemical approach and cation exchange.(1, 2)

For intercalation by decyl-trimethylammonium (C10), the wet $Ti_3C_2T_x$ sediment was soaked in 0.5 M of decyl-trimethylammonium bromide ($C_{13}H_{30}BrN$, 99%, TCI America) for 4 days at room temperature, with 2 min of daily shaking. The sample was then washed four times with deionized water without shaking, followed by vacuum-assisted filtration and an additional wash with 100 mL of deionized water.

For intercalation by hexadecylamine (HDA), the wet $Ti_3C_2T_x$ sediment was stirred in 0.5 M HDA ($C_{16}H_{35}N$, 95%, TCI America) at 75 °C for 24 h. The sample was then washed four times with deionized water, without shaking, followed by vacuum-assisted filtration, and an additional was with 100 mL of deionized water.

Material characterization

The structural morphology of the samples was characterized using scanning electron microscopy (SEM) with a ZEISS GEMINI 500 microscope equipped with an Xmax detector (Oxford Instruments). For imaging purposes and spectroscopic analysis, acceleration voltages of 2 kV and 15 kV, respectively, were employed. The samples were carefully mounted on aluminum stubs using copper tape, eliminating the need for additional conductive sputter coating.

High-resolution transmission electron microscopy was performed using a multi-purpose JEM-F200 microscope at an acceleration voltage of 200 kV. To prepare the samples, a copper grid coated with lacey carbon was used as the sample holder. Approximately 15 mg of the powder was dispersed in 15 mL of ethanol using an ultrasonic bath, and the sample was deposited dropwise onto a copper grid.

Phase analysis was performed using X-ray diffraction with a D8 Discover diffractometer (Bruker AXS) equipped with a Cu-Kα source (40 kV, 40 mA), a Göbel mirror, and a 1 mm point focus. A two-dimensional VANTEC detector captured an angular range of 20° 2θ for 1000 s, with measurements repeated three times to cover the 2-80° 2θ range. All scans underwent background subtraction and were normalized to (0,100). The system was calibrated using the NIST 1976b corundum standard.

Raman spectroscopy was conducted using a Renishaw inVia Raman microscope, which employed a neodymium-doped yttrium aluminum garnet laser with an excitation wavelength and power of 532 nm and 87 µW, respectively, at the focal point of the sample. We chose a magnifying lens with a numeric aperture of 0.75. Before obtaining the measurements, a silicon standard was used to calibrate. Each sample was placed on a glass slide, and spectra from six points were recorded with a 10-s exposure time and accumulated five times. We employed cosmic ray removal and normalized the intensity range to 0-100%.

The concentrations of the feed (50 mM LiCl+ 10 mM NaCl) and recovery (10 mM KCl) solutions were measured using inductively coupled plasma optical emission spectrometry (ICP-OES, ARCOS FHX22, SPECTRO Analytical Instruments). Samples (2 mL) were collected before and after the experiment and injected into the system at a flow rate of 1 mL/min. A correlation was established between the ion concentrations and corresponding signal intensities. Calibration was performed using mixed ion solutions with known concentrations of LiCl, NaCl, KCl, $CaCl_2$, and $MgCl_2$ at 1 µm, 5 µm, 0.1 mM, 0.5 mM, 1 mM, 5 mM, and 10 mM.(3)

To detect material variations in the chemical composition and oxidation states at the surfaces, X-ray photoelectron spectroscopy (XPS) was performed using the Kratos Axis Supra system (Kratos Analytical). For sample preparation, loose powders were sprinkled over the surface of a small piece of carbon tape (5 × 5 mm^2). The powders were then firmly pressed into the tape using the flat side of a freshly cleaned spatula. After the loose powder was removed from the sample surface, a holder was inserted into the XPS load lock. The photon source was a monochromatized Al Kα line. Survey spectra were recorded with an analysis area of 300 × 700 μm^2 and pass energy of 160 V at 15 kV and 10 mA. A pass energy of 80 eV was selected for acquiring the elemental spectra. The highly resolved measured spectra of Ti 2p and C 1s were recorded at a pass energy and power of 10 eV and 225 W, respectively. All spectra were analyzed using the CasaXPS software (version 2.3.15) and calibrated to the carbidic component in the C 1s signal (281.8 eV).

Thermogravimetric analysis was performed using a Netzsch TG-209-1 Libra system to analyze mass changes as the temperature increased to 1000 °C at a heating rate of 5 °C/min in an oxidative (synthetic air) atmosphere within an alumina crucible.

Electrode preparation

The electrodes were fabricated using vacuum filtration to ensure a uniform structure and optimal performance. MXenes were combined with carbon nanotubes (Nanocyl, NC7000) without the need for binder. The composition mass ratio of the electrodes was 9:1. The material was mixed with carbon nanotubes in ethanol and treated with a tip sonicator (Branson Sonifier 450) for 30 min to obtain a homogeneous mixture at 30% of the maximum

power output. After vacuum filtration, the electrodes were dried in an oven overnight at 60 °C under 6 kPa.

Electrochemical characterization

For electrochemical measurements of the cell, discs (30 mm) were punched from the prepared electrode sheets and used as working electrode. An oversized commercially available microporous activated carbon cloth (Kynol ACC-507-20) was used as the counter electrode (30 mm) in a flow-between electrochemical desalination cell with a flow rate of 5 mL/min.(3, 4) The electrodes were separated using a 26 mm round glass fiber filter (GF/A, Whatman). Key performance metrics are listed in *Supporting Information*, **Table S1**.

Electrochemical window opening measurements were performed using a BioLogic VMP-300 potentiostat/galvanostat in a potential window of -1 V to +1 V vs. Ag/AgCl at a scan rate of 0.1 mV/s. Galvanostatic charge/discharge with potential limitation measurements were conducted in the potential range of -0.8 V to +0.8 V with a holding time of 2 h. Cycling stability tests were performed using chronoamperometry at 0.6 V. All the electrochemical tests were conducted in a climate chamber held at 25±1 °C.

Calculations

The lithium ion extraction rate was calculated according to **Eq. (1)**:

$$Lithium\ ion\ extraction\ rate = \frac{\Delta C_{Li} \times \mathrm{V}}{\mathrm{t} \times \mathrm{A} \times \mathrm{M}} \qquad \textbf{(Eq. 1)}$$

where ΔC_{Li} is the concentration change of lithium ions, t is the time, A is the effective area of the electrode, M is the molar mass of lithium ions, and V is the volume of the solution.

The selectivity in terms of the lithium ion to sodium ion ratio was calculated according to **Eq. (2)**:

$$Selectivity = \frac{\Delta C_{Li}}{\Delta C_{Na}} \times \frac{C_{Na}\ \mathrm{initial}}{C_{Li}\ \mathrm{initial}} \qquad \textbf{(Eq. 2)}$$

where ΔC_{Li} and ΔC_{Na} represent the concentration (mg/L) changes of lithium ions and sodium ions cations in the solution, respectively.

Supporting Figures

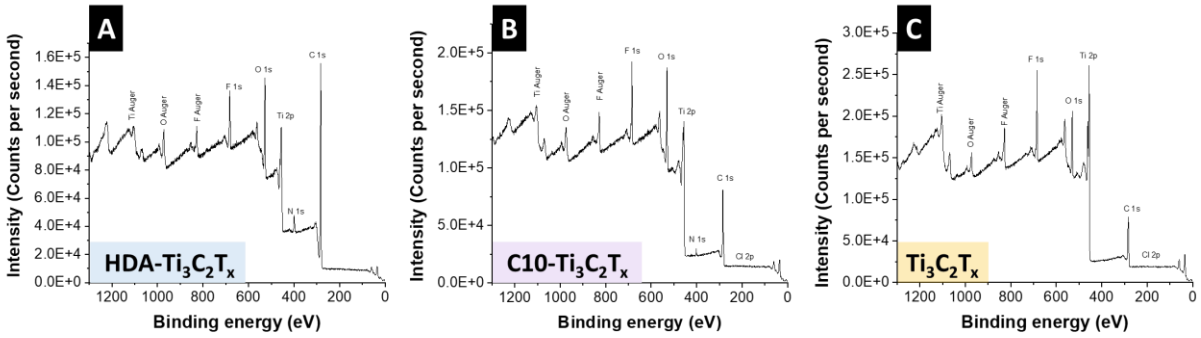

Figure S1. X-ray photoelectron survey spectra of the electrodes.

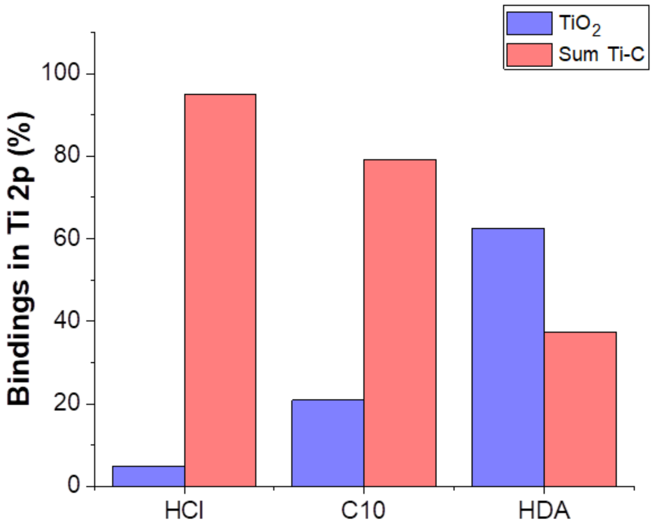

Figure S2. Binding values of the MXene materials.

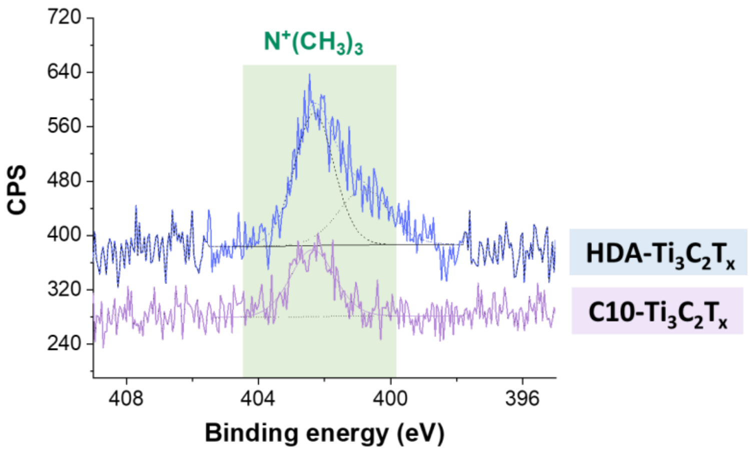

Figure S3. N 1s region in the X-ray photoelectron spectra for C10 and HDA intercalated $Ti_3C_2T_x$. CPS: counts per second.

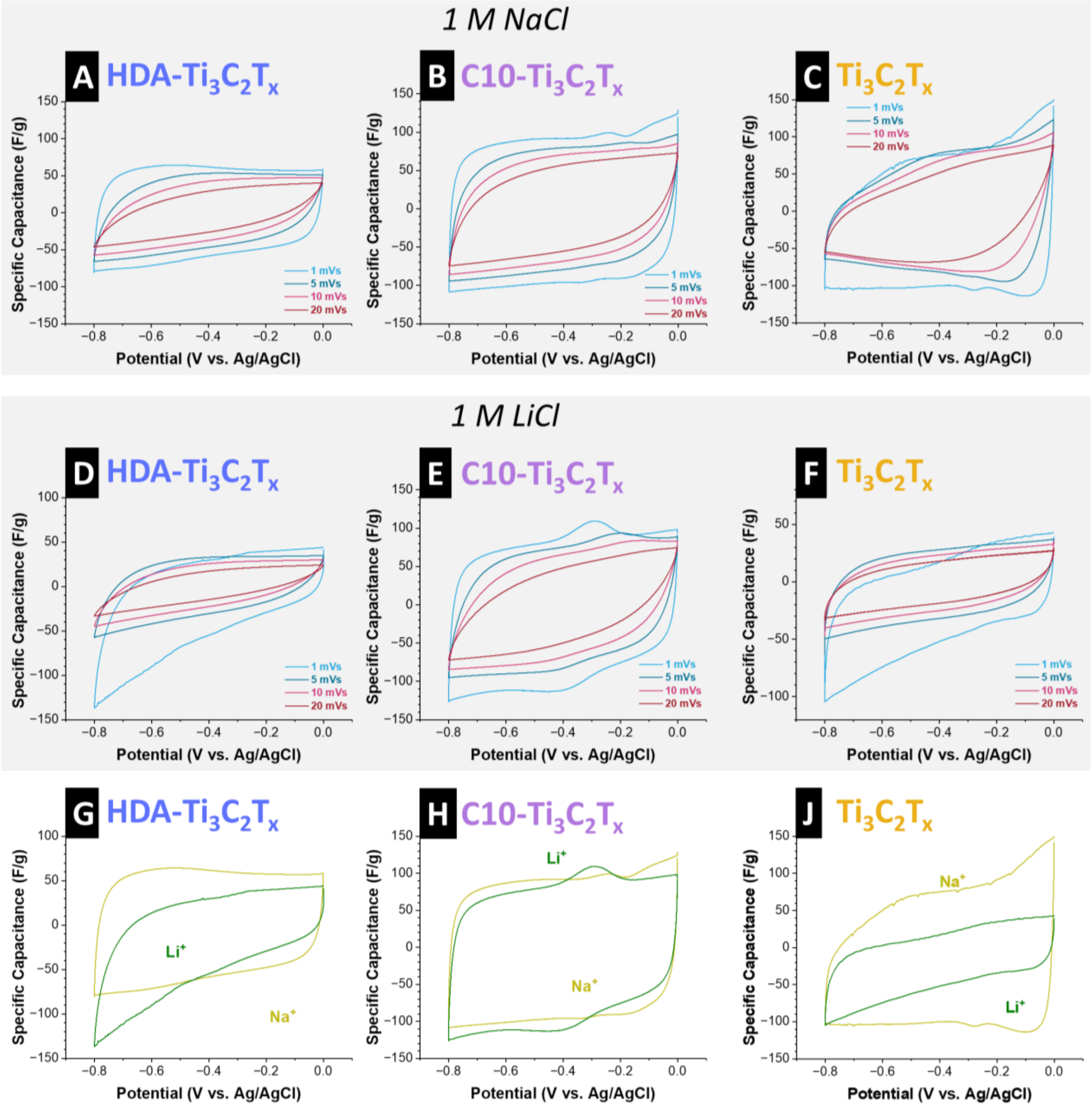

Figure S4. Cyclic voltammograms of the MXene electrodes in different scan rates in the range of 1 mV/s to 20 mV/s: A) HDA-$Ti_3C_2T_x$, B) C10-$Ti_3C_2T_x$, C) $Ti_3C_2T_x$, D) HDA-$Ti_3C_2T_x$, E) C10-$Ti_3C_2T_x$, F) $Ti_3C_2T_x$. Comparison of the electrodes in 1 M NaCl and 1 M LiCl in 1 mV/s: G) HDA-$Ti_3C_2T_x$, H) C10-$Ti_3C_2T_x$, J) $Ti_3C_2T_x$.

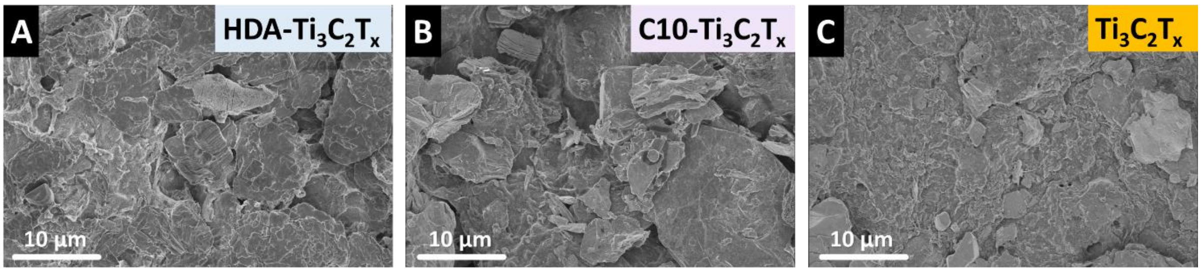

Figure S5. Post-mortem scanning electron micrographs of the electrodes.

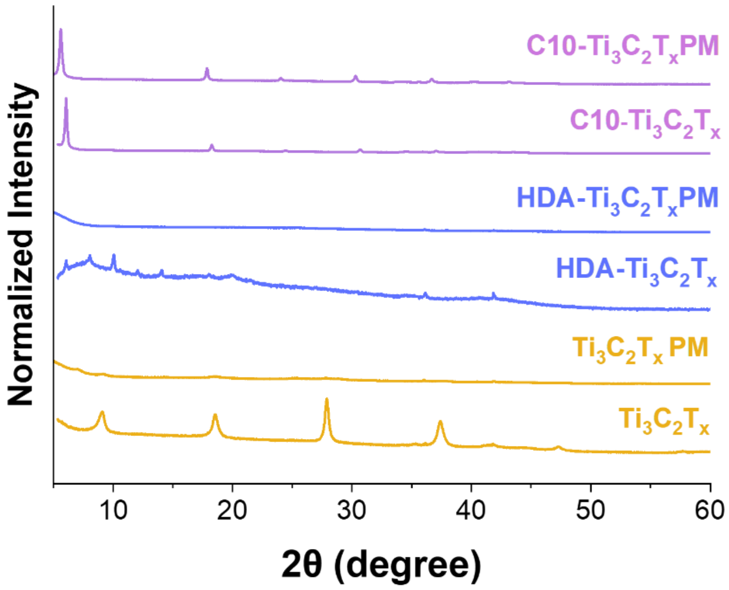

Figure S6. Post-mortem (PM) X-ray diffractograms of the electrodes.

Supporting Tables

Table S1: Data obtained from cyclic voltammetry.

	**Scan rate (mV/s)**	**Mass (mg)**	**Voltage window (ΔV)**	**Capacitance (F/g)**
HDA-$Ti_3C_2T_x$	1	16.69	0.8	37 F/g
C10-$Ti_3C_2T_x$	1	23.56	0.8	54 F/g
$Ti_3C_2T_x$	1	31.12	0.8	23 F/g

Table S2: Key performance metrics of the electrodes.

	**Electrode thickness (µm)**	**Mass loading (mg/cm²)**	**Conductivity (S)**	**Geometric area (cm²)**
HDA-$Ti_3C_2T_x$	290	21.9	12.4	1.13
C10-$Ti_3C_2T_x$	284	29.6	5.4	1.13
Pristine $Ti_3C_2T_x$	302	30.4	14.6	1.13

Supporting References

1. Liang K, Matsumoto RA, Zhao W, Osti NC, Popov I, Thapaliya BP, et al. Engineering the interlayer spacing by pre-intercalation for high performance supercapacitor MXene electrodes in room temperature ionic liquid. Advanced Functional Materials. 2021;31(33):2104007.
2. Lund A, Manohara GV, Song AY, Jablonka KM, Ireland CP, Cheah LA, et al. Characterization of Chemisorbed Species and Active Adsorption Sites in Mg-Al Mixed Metal Oxides for High-Temperature CO_2 Capture. Chemistry of Materials. 2022;34(9):3893–901.
3. Torkamanzadeh M, Kök C, Burger PR, Ren P, Zhang Y, Lee J, et al. Best practice for electrochemical water desalination data generation and analysis. Cell Reports Physical Science. 2023;4(11):101661.
4. Suss ME, Porada S, Sun X, Biesheuvel PM, Yoon J, Presser V. Water desalination via capacitive deionization: what is it and what can we expect from it? Energy & Environmental Science. 2015;8(8):2296–319.
